# Cerebrolysin in Patients with Subarachnoid Hemorrhage: A Systematic Review and Meta-Analysis

**DOI:** 10.3390/jcm12206638

**Published:** 2023-10-20

**Authors:** Klaudyna Kojder, Konrad Jarosz, Mateusz Bosiacki, Agata Andrzejewska, Sławomir Zacha, Joanna Solek-Pastuszka, Anna Jurczak

**Affiliations:** 1 Anesthesiology and Intensive Care Department, Pomeranian Medical University, 70-210 Szczecin, Poland; pastuszka@mp.pl; 2 Department of Specialist Nursing, Pomeranian Medical University, 70-210 Szczecin, Poland; jaroszki@interia.pl; 3 Department of Functional Diagnostics and Physical Medicine, Pomeranian Medical University, Zołnierska 54 Str., 71-210 Szczecin, Poland; bosiacki.m@gmail.com (M.B.); anna.jurczak@pum.edu.pl (A.J.); 4 Anesthesiology and Intensive Care Department, University Hospital 1, 72-252 Szczecin, Poland; november.rain@wp.pl; 5 Department of Pediatric Orthopedics and Oncology of the Musculoskeletal System, Pomeranian Medical University, 70-210 Szczecin, Poland; sekozacha@gmail.com

**Keywords:** SAH, cerebrolysin, neuroprotective treatment, meta-analysis

## Abstract

Subarachnoid Hemorrhage (SAH) is one of the acute neurological conditions that is associated with high mortality and recovery failure rates. In recent years, due to the development of endovascular and classical techniques, the mortality rate after SAH has decreased. Currently, more research is focused on understanding the molecular mechanisms underlying SAH. Methods of treatment are investigated in order to obtain the best treatment result, not only survival. One of the drugs used in stroke, including SAH, is Cerebrolysin. It is a mixture of neuropeptides that has similar properties to neurotrophic factors. Its positive impact on strokes has been analyzed; however, there are no meta-analyses concerning only the subpopulation of patients diagnosed with SAH in the current literature. Therefore, we conducted a meta-analysis of available clinical trials to evaluate the effect of Cerebrolysin on the treatment outcome. The data suggest a positive effect of Cerebrolysin on the mortality of SAH patients. However, further randomized clinical trials with larger groups of patients are needed to draw final conclusions.

## 1. Introduction

Aneurysmal Subarachnoid Hemorrhage (aSAH) is a type of stroke that is associated with high mortality and morbidity [1,2,3]. Subarachnoid Hemorrhage (SAH) is one of the acute neurological conditions that is associated with high mortality and recovery failure rates. The rupture of an intracranial aneurism is the most common cause of SAH. It is not only a large medical burden but also a big socio-economic burden as it affects predominantly middle-aged people of working age. The risk is also highlighted by asymptomatic aneurism carriers. According to Korja et al., it could be even 1 in 20 to 30 in the adult population. From those asymptomatic cases, approximately 25% will rupture [4,5].

Nontraumatic spontaneous SAH constitutes 1–7% of all strokes. According to the WHO MONICA stroke study, the fatality rate is above 30% for SAH [6]. In the latest report from the American Heart Association/American Stroke Association Guidelines (2023), the incidence of aSAH is ≈6.1 per 100,000 patients per year. Those data show that there is significant heterogeneity according to the incidences in different regions such as Finland and New Zealand [7,8,9]. In a report from China, aSAHs originated mostly from the anterior communicating artery (30.1%), then the posterior communicating artery (28.7%) and the middle cerebral artery (15.9%) [10]. The other origins of non-aneurysmal bleeding were as follows: brain arteriovenous malformation, Moyamoya disease, stenosis or sclerosis of the cerebral artery, and dural arteriovenous fistula or carotid cavernous fistula [10].

The risk factors that could be modified according to Feigin et al., as well as data from France and Audibert et al. (publication from 2005), are smoking, hypertension, and excessive alcohol use [11,12]. Other factors such as hormone replacement therapy, hypercholesterolemia, and diabetes remain unclear according to data from France and the USA. The risk factors listed there were as follows: female sex, race, hypertension, hyperlipidemia, cigarette smoking, cocaine use, excessive alcohol consumption, family history of SAH, and some connective tissue disorders [11].

Progress, both in classic neurosurgical approaches but also endovascular procedures, has reduced the early mortality rates. However, we are still aiming to improve the general outcome and the burden of delayed cerebral ischemia (DCI) [13]. DCI can affect patients in different time periods, although it usually happens in the first 2 weeks after the initial bleeding (4–10 days) [5]. DCI can occur in different ways: large vessel constriction, small vessel constriction, microthrombosis, free radical formation, and inflammation. Because of different origins, it seems reasonable to assume that there could be different targets for pharmacological treatment of DCI and primary injury repercussions [14]. Although DCI occurs in nearly 40% of all cases, it is responsible for about 50% of deaths in the SAH population as the delayed onset of events makes it more difficult to diagnose and to treat patients appropriately [5]. DCI also has a pathophysiology that is not yet completely understood. An important remark was pointed out in the work of Woo et al. that the delayed character of DCI gives us some time for reaction and treatment or prophylaxis [5]. However, only Nimodipine, a calcium channel blocker, is now proven to have a positive influence on SAH patients’ outcome by lowering the risk of DCI. This dihydropiridine derivative blocks the calcium influx to the smooth muscle cells of the vasculature, especially in the brain circuit. As a highly lipophilic substance, it easily accesses the brain–blood barrier. In that way, Nimodipine initiates the widening of vessels, positively affects cerebral blood flow, and protects brain cells from hypoxia. Nimodipine is either used in the USA or in Europe. It reduces calcium entry to smooth muscle cells and relaxes the arteries [13]. With regards to relaxing arteries, newer experimental and clinical approaches for detecting and treating DCI set the cerebral endothelium, neurovascular unit, and cortical spreading depressions, but also drugs such as lithium for calcium stabilization, into focus [15,16,17,18,19,20].

Other currently used pharmacological agents include Fasudil, registered in Japan and China—a rho kinase inhibitor, which inhibits smooth muscle contraction. Another agent used in Japan for SAH treatment is OKY-046, a platelet aggregation inhibitor [21]. Other agents investigated are statins, magnesium, sodium nitrate, and Cerebrolysin.

Cerebrolysin is a mixture of peptides isolated from purified porcine brain tissue. The cerebroprotective properties of Cerebrolysin are associated with the short-chainneurotrophic factors contained within the agent, expressing, among others, anti-inflammatory abilities. Cerebrolysin also reduces astrogliosis and enhances neuroneogenesis in in vitro and in vivo studies. It has been shown to have beneficial results for traumatic brain injury (TBI), stroke, and also with SAH patients [14,22]. The way Cerebrolysin interacts with several neurotrophic factors shows its multimodal character [23]. It has also been observed that Cerebrolysin acts with the tumor necrosis factor alpha TNF-α, the nerve growth factor (NGF), the Insulin-like growth factor (IGF-1), the brain-derived NTF (BDNF), and the vascular endothelial growth factor (VEGF) [13]. Those factors influence each other and the molecular paths of Caspase-3 and T-cells. Influencing and targeting many points shows its multimodal activity in neuroprotection, which is used in stroke but also in TBI and chronic neuro diseases like dementia [23]. As in TBI, it has also been proven in stroke that Cerebrolysin acts at the Sonic Hedgehog Signaling Pathway (SHH)—one of the crucial pathways for neural cell restoration and neuroneogenesis. In their work with rodents, the authors proved that, after Cerebrolysin exposure, neural progenitor cells are activated via the SHH pathway [24]. Cerebrolysin influences the glutaminergic, GABAergic, and cholinergic pathways. In stroke, the positive role of Cerebrolysin has been proven in randomized placebo-controlled clinical trials. Cerebrolysin improves the National Institutes of Health Stroke Scale (NIHSS), the modified Rankin Scale (mRS), and the Barthel Index (BI) 3 months after the initial stroke [5]. In a double-blind placebo-controlled randomized trial for patients with Acute Ischemic Stroke diagnosis performed in Asia—CASTA—a total of 1070 patients were included. Cerebrolysin positively affected the severely damaged patients involved in the trial [24,25].

The safety of Cerebrolysin for patients diagnosed with stroke has been already analyzed in a meta-analysis from 2021 [26]. However, according to our knowledge, there are no meta-analyzed data concerning Cerebrolysin efficiency in stroke or SAH. In our research, we performed a meta-analysis of clinical trials with patients diagnosed with SAH, treated with Cerebrolysin intravenously. We tried to analyze the data including GCS, GOS, LOS, and mortality of patients.

## 2. Materials and Methods

### 2.1. Search Strategy

Two independent authors (KK and KJ) searched the following databases: Pub Med, Cinahl, Web of Science, and Embase. The search was performed from database inception until 11 July 2022, and the only restriction was English as the publication language. The search included studies evaluating the effect of Cerebrolysin in the treatment effect of patients with a SAH diagnosis. The search strings are listed in Supplementary Table S1.

A manual review of relevant and criteria-meeting publications was also applied to the reference list from eligible publications. The inclusion criteria were human studies, adult patients (>18 years), diagnosis of SAH, and Cerebrolysin treatment. The exclusion criteria were animal studies, in vitro studies, reviews, systematic reviews, editorials, individual case reports, opinions, editorials, studies including pediatric patients (<18 years), and studies in any language other than English.

### 2.2. Data Abstraction

Data on the study design, patient characteristics, and Cerebrolysin treatment were independently extracted from each study in accordance with the Preferred Reporting Items for Systematic Reviews and Meta-Analyses (PRISMA) standard [27]. However, the study was not registered in accordance with the statement in the PRISMA checklist (2020) [27] that registration is not obligatory. As outcomes, the Glasgow Outcome Scale (GOS), Glasgow Coma Scale (GCS), mortality, and Length of Stay (LOS) were collected. Two independent investigators (KJ and KK) prepared the data.

### 2.3. Statistical Analysis

We conducted a random-effects meta-analysis of outcomes for which ≥2 studies contributed data, using Comprehensive Meta-Analysis V3 [28]. Study heterogeneity was determined using the chi-square test of homogeneity, with *p* < 0.05 indicating significant heterogeneity. All analyses were two-tailed with the alpha equal to 0.05.

For continuous outcomes, we analyzed the standardized differences in the means of endpoint scores using observed cases (OCs). Categorical outcomes were analyzed by risk ratio (RR). We aimed to conduct subgroup and exploratory maximum likelihood random-effects meta-regression analyses of the co-primary outcomes (e.g., with age, sex); however, due to insufficient data, we were not able to conduct this. Finally, we inspected funnel plots and used Egger’s regression test as well as Duval and Tweedie’s trim and fill method to quantify whether publication bias could have influenced the results [29].

### 2.4. Risk of Bias

The quality of each study methodology was classified using the Newcastle—Ottawa Quality Assessment Scale adapted for cross-sectional, cohort, and case-control studies. For selected studies, the following criteria were assessed: selection, comparability, and assessment of outcome. We assumed that “Low risk of bias” determines the study to be at low risk of bias for all domains; “Some concerns”—the study is judged to raise some concerns in at least one domain for this result but not to be at high risk of bias for any domain; “High risk of bias”—the study is judged to be at high risk of bias in at least one domain for this result, or the study is judged to have “some concerns” for multiple domains in a way that substantially lowers confidence in the result. The maximum star rating was 9. Less than 5 stars indicated low quality, 5–7 stars indicated moderate quality, and 8–9 stars indicated high quality.

### 2.5. The Quality of Studies

None of the four cohort studies achieved an overall “Low risk of bias” rating across all domains evaluated. Three studies in summary achieved 5–7 stars, indicating moderate quality. One study achieved 9 stars in total summary, which indicates the high quality of the study methodology. The results are presented in Supplementary Table S2.

## 3. Results

### 3.1. Search Results

**Figure 1:**
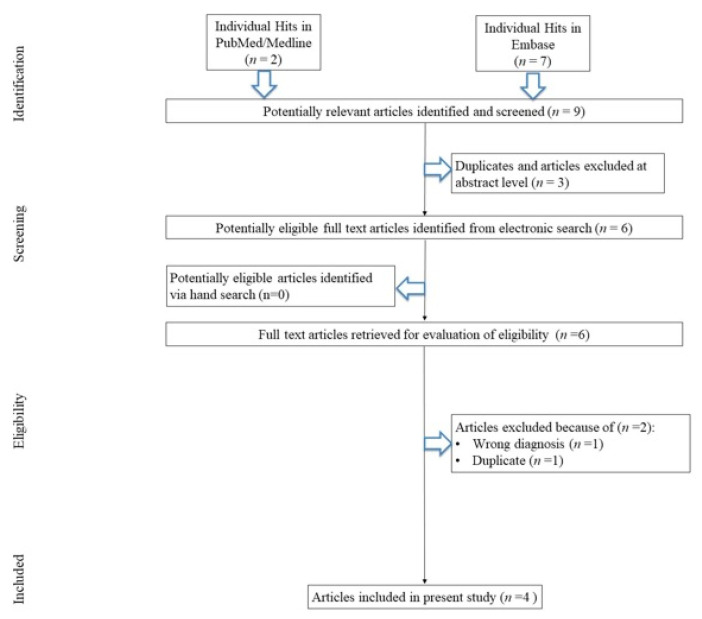
Study flow chart.

The initial search yielded nine hits. There were three studies which were excluded for being duplicates and/or after evaluation on the title/abstract level. No additional article was identified via hand search. Then, six full-text articles were reviewed. Of those, two were excluded due to not fitting the inclusion criteria. Reasons for exclusion were TBI diagnosis (n = 1) and duplication (abstract; n = 1) (Figure 1), yielding four studies that were included in the meta-analysis.

### 3.2. Study, Patients, and Treatment Characteristics

Altogether, four studies (n = 530) were included and are presented in Table 1 The intervention was administered to patients for 6–21 days, with a total study duration of up to 6 months. In all studies, patients were given Cerebrolysin intravenously at a dosage of either 30 or 50 mL/day. The age range of patients was 52–65 years. And the percentage of males varied between 28 and 40%. Characteristics are displayed in Table 2 below.

Some of the patients were qualified for classic neurosurgical treatment or additional craniotomy (up to 72% in the trial). Some of the patients were qualified and underwent an end-vascular procedure, i.e., coiling (up to 31.8%). The status of the patients reported in the analyzed papers was divided in Hunt–Hess classification and Fisher’s classification as listed in the Table 2. Because of the diversity of patients reported by Park YK et al., those data were divided according to the SAHs’ severity grade, as demonstrated in Table 1 and Table 2 below [30]: poor- and good-grade SAH—Park YK 1, good-grade SAH (HH ≤ 2)—Park YK 2, poor-grade SAH (HH ≥ 3)—Park YK 3.

### 3.3. Outcomes

The following data were abstracted from the studies: the Glasgow Coma Scale score (GCS), Length of Stay (LOS), and mortality.

### 3.4. Effect of Cerebrolysin on LOS

We were not able to meta-analyze the results on the effect of Cerebrolysin on LOS because of insufficient data. However, concerning the median value, it could be noticed that the control groups were hospitalized for longer in three of the five reports. There is a need for further studies in this matter. All the data extracted from reports are presented in Table 3 and Table 4 below.

### 3.5. Effect of Cerebrolysin on Mortality

Using random-effects analysis, the risk ratio for mortality in patients treated with Cerebrolysin compared to non-interventional arms was 0.525, with a 95% confidence interval of 0.279 to 0.846. The heterogeneity was low (Q value = 1.053; df = 2; *p* = 0.59; I2 = 0). Results are placed in Supplementary Figure S1

Egger’s test did not suggest a publication bias regarding the net effect of Cerebrolysin on mortality (Egger’s test: *p* = 0.457;—DM: *p* = 0.007; Supplementary Figure S2).

No other variables were analyzed due to insufficient data. However, abstracted data are placed in Table 4 above (GCS, GOS, LOS, and mortality).

### 3.6. Limitations of the Study

The limitations of the presented meta-analysis result, among other factors, from the relatively small number of reported studies. Most reports regarding the effect of Cerebrolysin on treatment outcomes focus on the effect in the entire population of stroke patients. There are fewer reports on the SAH subpopulation. Analyzed reports describeheterogeneous groups of patients in terms of the severity of the disease (Hunt–Hess, Fisher scale), different drug dosages, and different times of drug introduction, leading to heterogenous results in terms of outcome. We also could not identify additional but important data concerning, for example, DCI, which is one of the important risk factors of mortality in patients with SAH. The similar aspects of the analyzed studies were the age and sex of patients.

## 4. Discussion

Cerebrolysin has been investigated in stroke, and several trials have shown its beneficial role for improving functional outcome. Although a meta-analysis regarding Cerebrolysin in stroke can be found in the literature, there is a lack of data summarizing the effects concerning only SAH patients [9].

Our meta-analysis compares the treatment results among the clinical studies found in Pub Med and Embase from database inception until 11 July 2022, describing the effect of Cerebrolysin on 530 patients diagnosed with SAH. The main finding in our meta-analysis was an association between treatment with Cerebrolysin and lower mortality. We were not able to analyze the effect of Cerebrolysin on the outcome in terms of GOS and LOS because of insufficient data.

The limitations of this meta-analysis include the lack of large randomized trials, different doses of the drug administered at different time points after primary hemorrhage, the heterogeneous group of respondents, and heterogeneous results in terms of outcome. Therefore, it was not possible to compare all the publications mentioned in all planned aspects.

The heterogeneity of the groups results from the diversity of individual publications regarding the population, the inclusion and exclusion criteria, the severity of injury, the type of study, and the study methodology. The analyzed data included 30 mL (Hong Kong) and 50 mL (Poland) dosages as well as a 6–21-day range of administration time [5,31].

In summary, the study of Park et al. concludes that there is a potential role of Cerebrolysin in reducing the mortality rate in patients with SAH [30]. The work of Woo concentrated on cognitive performance after a 6-month period after initial trauma, concluding that Cerebrolysin is safe as an add-on therapy, but the results of cognitive assessment did not differ from those without Cerebrolysin administration [5].

The results of the analyzed works, e.g., the impact on mortality, may be the result of the influence of Cerebrolysin presented in the field of basic sciences. The possible mechanisms of cerebral impairment and secondary injury, especially concerning DCI, could involve vascular constriction and microthrombosis. The other pathologies mentioned in the literature are as follows: cortical spreading depolarization, blood–brain barrier breakdown, cerebral autoregulation impairment, and neuroinflammation [9,33].

Cerebrolysin has been investigated in molecular studies and has been proven in cellular experiments as a drug that decreases microglial activity, excitotoxicity, and the production of free radicals [34,35]. Cerebrolysin reduced changes in blood–cerebrospinal fluid barrier permeability after traumatic brain injury in a study involving rodents by Sharm et al. [34]. Those features could have a positive influence on the outcome in terms of mortality, as shown in our meta-analysis.

In the pathology of SAH, there are different pathways leading to neuronal cells: neuronal necrosis, neuronal programmed cell death (apoptosis), pyroptosis, and necroptosis, as described shortly below. Neuronal necrosis, as a passive process, does not consume energy. However, it is one of the most important path ofneuronal cell death in early brain injury in SAH [36]. The processes involved in this non-planned cell death are changes in cell membrane permeability, cell oedema, nuclear changes, DSA dissolution, and calcium and sodium ion accumulation within the intracellular compartment, which coexists with potassium outflow [35].

Neuronal programmed cell death includes apoptosis in pathways directed via numerous exogenous death receptors (rec TGR5, EGFR, TNFa, FasL, Trail) and in endogenous mitochondrial pathways [35]. The endogenous pathway starts with the Bcl-2 gene family, which enables mitochondrial membrane permeability through releasing cytochrome C, which binds with apoptosis protease activating factor 1 (Apaf-1) [26]. This complex process initiates apoptosis through the Caspase-3 route [36]. The external factor activates the previously mentioned receptors, and the following pathways are activated: AMP/PKCε/ALDH2 via TGF5 (in rats) [37], src/EGFR/STAT3 via receptor 30 (GPR 30), [38], Caspase-3 inhibition via statin receptors [36], PPARγ, and Bcl-2 expression, minimizing the factors Bax and NF-κB via Netrin 1 [36], ROS/ASK1/P38 via PDK4 expression [39], and Notch1/ASK1/p38 MAPK via fluoxetin administration [40]. All those factors could be beneficial in inhibiting pro-apoptotic evolution. Neuronal pyroptosis is a new term introduced in 2001 which also ends with cell death but is mainly associated with inflammation processes [40].

Neuronal necroptosis is a caspase-independent programmed cell apoptosis which involves serine-threonine kinase 3 (RIPK3) and the mixed lineage kinase domain (MLKL) [41]. In those pathways, TNFalfa-induced cell death is the most known.

One of the discovered features of Cerebrolysin is its anti-apoptotic role in the damaged brain [42,43]. It can influence the proteolytic pathways, and in observational studies, it reduced the number of apoptotic neurons after experimental glutamate exposure [42]. In their work, Formichi et al., in 2012, discovered the protective role of Cerebrolysin in spontaneous and induced apoptosis on peripheral blood lymphocytes in vitro [42]. In TBIs, Cerebrolysin reduces neuronal death and neuroinflammation via the TRL receptor in neuronal models [44]. In recent work from June 2023, the authors report that Cerebrolysin reduces the expression of Calpain and Caspase-3. It might have a positive influence on patients with SAH, considering the aforementioned study performed on rodents after ischemic reperfusion [45].

Cerebrolysin also shows its protective properties in reducing oxidative stress and lipid peroxidation. Avci et al., their 2022 study on primary cortex neurons, also showed the antioxidant and also abilities of lowering the inflammatory cytokine level of Cerebrolysin [46]. They also indicated a positive effect on glutamate transporters, which also plays a role in neurotoxicity leading to cell death [46].

The molecular changes underlying brain cell death in SAH are complicated and unclear. The pathways leading to cell death are complex, and many mechanisms of necrosis, apoptosis, autophagy, and necroptosis are present in the cell death pathology of SAH [34]. Cerebrolysin, according to the mentioned literature, might work with at least some of those pathways, protecting the brain from secondary damage during SAH and DCI trauma and lowering the mortality as the outcome.

The cerebroprotective mechanisms in which Cerebrolysin participates at the molecular and tissue levels are partly reflected in the results of the mentioned clinical trials. Perhaps it is the many points of targeting, including the impact on blood–brain barrier integrity, anti-apoptotic properties, and anti-inflammatory action, that enable Cerebrolysin to influence the mortality rate in patients with SAH. More randomized research is certainly needed to confirm these conclusions. However, these results make it reasonable to consider the use of Cerebrolysin in patients with SAH in clinical practice.

## 5. Conclusions

Our meta-analysis may indicate positive treatment effects of Cerebrolysin on clinical outcomes: specifically, the mortality rate of patients suffering from SAH. We were not able to analyze the effect of Cerebrolysin on GOS because of insufficient data. Cerebrolysin, however, might positively influence the LOS of patients diagnosed with SAH. Therefore, more randomized studies with larger, more homogenous groups of patients are needed.

## Figures and Tables

**Table 1:** Surgery qualifications and admission status of patients. HH—Hunt–Hess classification, F—Fishers classification, ND–no data.

Surgical Approach			HH 1,2		HH 3,4,5		F 1,2		F 3,4	
Reference	Craniectomy/Clipping (n)	Endovascular (n)	C−	C+	C−	C+	C−	C+	C−	C+
Park YK et al. 1 [30]	328	134	151	65	177	69	36	17	292	117
Park YK et al. 2 [30]	148	68	151	65	-	-	29	11	122	54
Park YK et al. 3 [30]	180	66	-	-	177	69	7	6	170	63
Woo PYM et al. [5]	15	35	ND	ND	ND	ND	11	6	13	19
Kojder K et al. [31]	3	5	1	0	4	3	0	0	5	5
Poljakovic et al. [32]	ND	ND	0	10	0	0	ND	ND	ND	ND

**Table 2:** Study and patient characteristics. ND—no data, PBO—placebo.

Study Characteristics						Intervention	Comparator	Sample Characteristics	
Reference	Country	Sponsorship	Blinding (Y/N)	Trial Duration (Days)	N Total Analyzed	Cerebrolysin Mean Dose/Day (mL)	PBO or Other Intervention	Age (Mean)	% Male
Park YK et al. 1 [30]	Republic of Korea	No	No	10–20	462	30	*-*	55	34
Park YK et al. 2 [30]	Republic of Korea	No	No	9–17	216	30	*-*	54	34
Park YK et al. 3 [30]	Republic of Korea	No	No	12–21	246	30	*-*	56	34
Woo PYM et al. [5]	Hong Kong	No	Yes	10–21	50	30	*-*	53	28
Kojder K et al. [31]	Poland	No	No	6–21	8	50	*-*	61	40
Poljakovic et al. [32]	Croatia	No	No	ND	10	ND	*-*	ND	ND

**Table 3:** The LOS according to references and Cerebrolysin supply.

Variable	Length of Stay							
	Cerebrolysin				Control			
Reference	Median	Range	SD	n	Median	Range	SD	n
Park et al., 2018 [30]	18	[15.0; 26.0]	ND	134	22.0	[17.0; 34.0]	ND	328
Park et al., 2018 [30]	33.0	[16.0; 70.0]	ND	65	30.0	[15.0; 67.0]	ND	151
Park et al., 2018 [30]	23.0	[16.0; 15.5]	ND	69	27.0	[17.0; 60.0]	ND	177
Woo et al., 2020 [5]	33	ND	15	25	39	ND	32	25
Kojder et al., 2020 [31]	9	3–22	ND	5	13	3–28	ND	5
Poljakovic, et al., 2022 [32]	ND	ND	ND	10	ND	ND	ND	ND

**Table 4:** GCS and mortality according to references and Cerebrolysin supply.

Reference	GCS C− [Mean]	GCS C+ [Mean]	Mortality C−	Mortality C+
Park YK et al. 1 [30]	13.0	14.0	57 (17.4%)	12 (9%)
Park YK et al. 2 [30]	15.0	15.0	12 (7.9%)	6 (9.2%)
Park YK et al. 3 [30]	11.0	8.0	45 (25.4%)	6 (8.7%)
Woo PYM et al. [5]	ND	ND	4 (16%)	0
Kojder K et al. [31]	8.2	6	2 (40%)	1 (20%)
Poljakovic et al. [32]	ND	ND	-	3 (30%)

## Data Availability

All the additional data are to be provided upon contact with corresponding author.

## Supplementary Materials

The following supporting information can be downloaded at: https://www.mdpi.com/2077-0383/12/20/6638/s1, Table S1. Strings used in databases search. Table S2. The risk of bias according to Newcastle—Ottawa Quality Assessment Scale. Figure S1. Effect of Cerebrolysin on mortality (Z value = −1.596, *p* = 0.012). Figure S2. Funnel plot for mortality (RR) in present meta-analysis.
