# Supplementary material for: Cerebrolysin in Patients with Subarachnoid Hemorrhage: A Systematic Review and Meta-Analysis
Source: J Clin Med. 2023 Oct 20;12(20):6638. doi: 10.3390/jcm12206638 (PMC10607250; doi:10.3390/jcm12206638)
Supplement: Supplementary file 1 [file jcm-12-06638-s001.zip › jcm-2611228-supplementary.pdf]

Supplementary Table S1.-Strings used in databases search

Database	Search Strings with Medical Subject Headings
**Pub Med./Cinahl/Web Of Science**	(subarachnoid hemorrhage OR aneurysmal subarachnoid haemorrhage OR aneurysmal subarachnoid hemorrhage OR arachnoid haemorrhage, brain OR arachnoid hemorrhage, brain OR arachnoidal bleeding OR arachnoidal haemorrhage OR arachnoidal haemorrhage, brain OR arachnoidal hemorrhage OR arachnoidal hemorrhage, brain OR bleeding, subarachnoid OR brain arachnoid haemorrhage OR brain arachnoid hemorrhage OR haemorrhage, subarachnoid OR hemorrhage, subarachnoid OR spontaneous subarachnoid haemorrhage OR spontaneous subarachnoid hemorrhage OR subarachnoid bleeding OR subarachnoid blood OR subarachnoid haematoma OR subarachnoid haemorrhage OR subarachnoid haemorrhage, brain OR subarachnoid haemorrhage, traumatic OR subarachnoid hematoma OR subarachnoid hemorrhage OR subarachnoid hemorrhage, brain OR subarachnoid hemorrhage, traumatic OR subarachnoid hemorrhagia OR subarachnoidal bleeding OR subarachnoidal haemorrhage OR subarachnoidal hemorrhage OR traumatic subarachnoid haemorrhage OR traumatic subarachnoid hemorrhage) AND (cerebrolysin OR cerebrolysin OR cerebrolysine) AND (glasgow outcome scale OR rankin scale OR mini mental state examination OR mortality OR barthel index OR barthel adl index OR barthel index OR montreal cognitive assessment score)
**Embase**	('subarachnoid hemorrhage' OR 'aneurysmal subarachnoid haemorrhage' OR 'aneurysmal subarachnoid hemorrhage' OR 'arachnoid haemorrhage, brain' OR 'arachnoid hemorrhage, brain' OR 'arachnoidal bleeding' OR 'arachnoidal haemorrhage' OR 'arachnoidal haemorrhage, brain' OR 'arachnoidal hemorrhage' OR 'arachnoidal hemorrhage, brain' OR 'bleeding, subarachnoid' OR 'brain arachnoid haemorrhage' OR 'brain arachnoid hemorrhage' OR 'haemorrhage, subarachnoid' OR 'hemorrhage, subarachnoid' OR 'spontaneous subarachnoid haemorrhage' OR 'spontaneous subarachnoid hemorrhage' OR 'subarachnoid bleeding' OR 'subarachnoid blood' OR 'subarachnoid haematoma' OR 'subarachnoid haemorrhage' OR 'subarachnoid haemorrhage, brain' OR 'subarachnoid haemorrhage, traumatic' OR 'subarachnoid hematoma' OR 'subarachnoid hemorrhage' OR 'subarachnoid hemorrhage, brain' OR 'subarachnoid hemorrhage, traumatic' OR 'subarachnoid hemorrhagia' OR 'subarachnoidal bleeding' OR 'subarachnoidal haemorrhage' OR 'subarachnoidal hemorrhage' OR 'traumatic subarachnoid haemorrhage' OR 'traumatic subarachnoid hemorrhage') AND ('cerebrolysin' OR 'cerebrolysin' OR 'cerebrolysine') AND ('glasgow outcome scale' OR 'rankin scale' OR 'mini mental state examination' OR 'mortality' OR 'barthel index' OR 'barthel adl index' OR 'barthel index' OR 'montreal cognitive assessment score')<br>')

Supplementary Table S2. The risk of bias according to Newcastle—Ottawa Quality Assessment Scale.

	Type of study		Selection bias - summary (max. 4 stars)	Comparability<br>- summary (max. 2 stars)	Outcome – summary (max. 3 stars)	Total numer of stars (max. 9 stars)
Park YK et al. [30]	Cohort	Retrospective cohort	3	2	2	7
Woo PYM et al. [5]	Cohort	Randomised, prospective cohort study	4	2	3	9
Kojder K et al [31]	Cohort	Retrospective cohort	3	2	1	6
Poljakovic et al. [32]	Cohort	Retrospective cohort	2	2	1	5

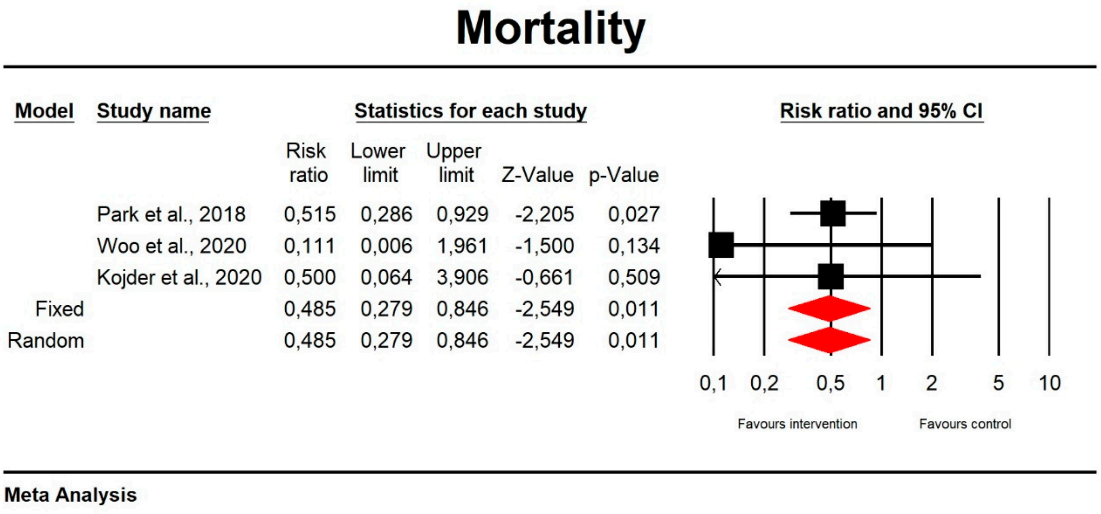

Supplementary Figure S1. Effect of Cerebrolysin on mortality (Z value=-1.596, p=0.012)

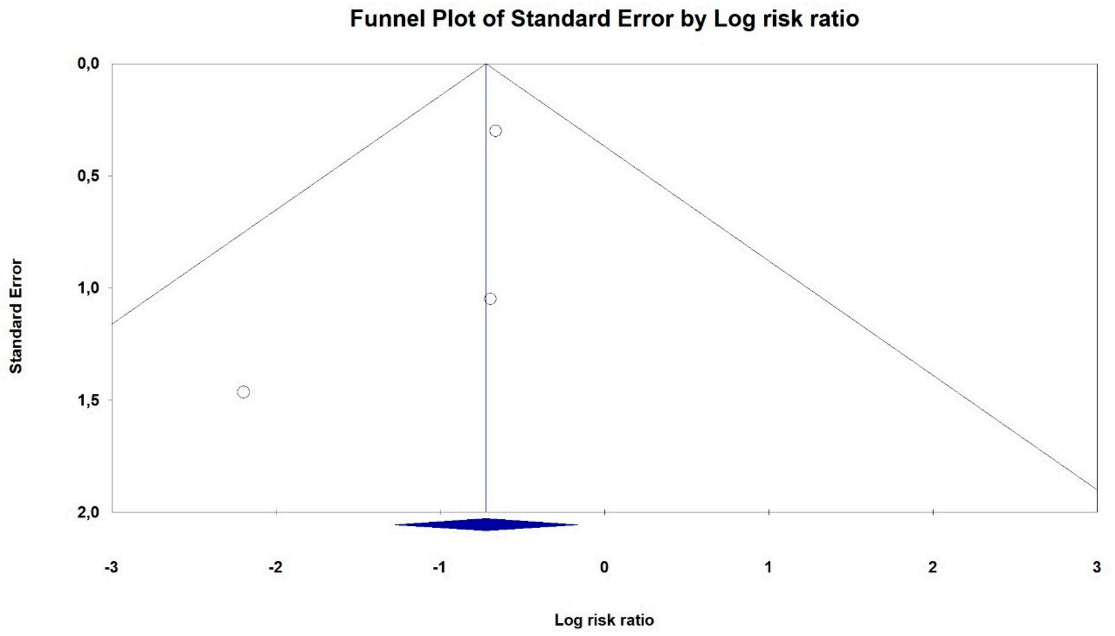

Supplementary Figure S2. Funnel plot for mortality (RR) in present meta-analysis.
